# Reducing the risk of prenatal alcohol exposure and FASD through social services: promising results from the FAR SEAS pilot project

**DOI:** 10.3389/fpsyt.2023.1243904

**Published:** 2023-09-15

**Authors:** Katarzyna Okulicz-Kozaryn, Lidia Segura-García, Carla Bruguera, Fleur Braddick, Marta Zin-Sędek, Claudia Gandin, Luiza Słodownik-Przybyłek, Emanuele Scafato, Silvia Ghirini, Joan Colom, Silvia Matrai

**Affiliations:** ^1^Department of Children and Adolescents Health, Institute of Mother and Child (IMiD), Warsaw, Poland; ^2^Subdirectorate General of Addictions, HIV, STI and Viral Hepatitis, Public Health Agency of Catalonia (GENCAT), Barcelona, Spain; ^3^CLÍNIC Foundation for Biomedical Research (FCRB), Barcelona, Spain; ^4^National Centre for Prevention of Addictions (KCPU), Warsaw, Poland; ^5^Istituto Superiore di Sanità (ISS), Rome, Italy

**Keywords:** fetal alcohol spectrum disorder (FASD), prenatal alcohol exposure, prevention, intervention, local community

## Abstract

**Introduction:**

Within FAR SEAS, a multi-component evidence-based community intervention was implemented and evaluated in Mazovia (Poland), with the aim of preventing alcohol-exposed pregnancies, and therefore preventing FASD.

**Methods:**

Multi-disciplinary professionals from different services (social, addiction, and psychology), recruited women of child-bearing age (pregnant and not pregnant) in local communities, screened them for alcohol risk, and allocated participants (*n* = 441) to groups for low- (70%), moderate- (23%), or high-risk (7%) of alcohol exposed pregnancy, to provide interventions tailored to their needs. The non-parametric sign test, testing differences between pairs of observations before and after intervention was used to evaluate the outcomes.

**Results:**

Follow-up data (collected from 93% of participants) indicated positive changes in the key outcome variables: risky alcohol consumption dropped by 81%, contraception use increased by 15% and visiting a gynecologist increased by 39%; as well as in associated psychosocial risk factors (decrease in cigarette and drug use, domestic violence and depressive symptoms). No changes were noted in frequency of other service use (medical, psychological, or social). The most prominent changes were observed in the moderate-risk group.

**Discussion:**

Changing risky behaviors (alcohol consumption and sex without contraception) to prevent alcohol exposed pregnancies is feasible at the local level, even without engagement of medical professionals. Key challenges, related to engaging professionals and local authorities, must be addressed; and procedures should be adapted to local contexts and needs.

## 1. Introduction

Alcohol consumption during pregnancy may result in a series of adverse effects to the fetus including congenital anomalies and behavioral, cognitive and adaptive deficits, collectively known as Fetal Alcohol Spectrum Disorder (FASD). FASD is preventable by abstaining from drinking alcohol during pregnancy but effective prevention is complex and requires activities at various levels, targeting general population, women of childbearing age, women with alcohol problems and postpartum (1). For this reason, the EU strategy to support Member States in reducing alcohol related harm (2) re-quested governments to raise awareness of the risks of drinking during pregnancy, and stressed the need for evidence-based policies and practices to reduce alcohol related harms. In line with this statement, the European Commission awarded a service contract under the 2018 EU health program to deliver a project called FAR SEAS, with the aim of promoting European knowledge exchange, and piloting regional strategies to reduce FASD. The main objectives of FAR SEAS were to promote regionally implemented strategies to reduce and prevent fetal alcohol syndrome (FAS) and fetal alcohol spectrum disorder (FASD); and to facilitate knowledge-exchange and capacity-building among EU Member States. One of the key elements of the project was to test the implementation of a multi-component, evidence-based, community intervention aimed at preventing alcohol consumption among pregnant women and women in child-bearing age, through a regional-level pilot study.

The region chosen for the pilot was the Mazovian voivodeship in central Poland due to: relatively high prevalence of alcohol consumption reported by Polish pregnant women (from 15 to 39%, depending on the study) (3); significant FASD prevalence among Polish school students aged 7 to 9 years, in line with the estimated prevalence of FASD in the entire WHO European region (4) [the Polish prevalence is estimated to be higher than 20 cases per 1000; (5)].

The piloted program aimed to reduce the risk of prenatal alcohol exposure (PAE) in the general population of women of childbearing age. Specific objectives included:

• Reduction of risky alcohol consumption (among not pregnant women) and prevention of alcohol use among pregnant women;
• Increasing effective contraception use (among not pregnant women who drink alcohol), given that a high risk of fetal alcohol exposure is associated with unplanned pregnancy. Since a majority of women drink alcohol regularly (at least once a month), the likelihood that they will continue drinking until they find out they are pregnant is very high (6). Research indicates that about 30% of pregnancies in Poland are unplanned (7, 8);
• Increasing use of professional support to deal with complex psychological, medical and social challenges, which have been found to increase the risk of alcohol use during pregnancy.

Based on the literature review and consultation of Polish experts in the FAR SEAS project, depression was selected as one of the key factors to be addressed. The lifetime prevalence of depression among Polish women from 18 to 49 years of age is 4% (9). Systematic reviews have shown that about 10% of pregnant women and 13% of those who have given birth experience some type of mental disorder, most commonly depression or anxiety (10, 11). Although the estimates of the prevalence of depression during pregnancy vary widely, ranging from 0.5 to 51%, rates of depression, especially during the second and third trimesters of pregnancy, are substantial (12). Moreover, women with depression have shown greater difficulty in changing their behavior and reducing alcohol consumption during pregnancy than women without depression (13).

Intimate partner violence is another factor associated with a higher risk of alcohol consumption during pregnancy (14–17). Research showed that 27% of women in Poland experienced domestic violence at least once (18). Women who experience violence live in unpredictable, hard-to-control and difficult to manage environments, which may impede their efforts to reduce drinking and practice birth control. They may also be at higher risk of using alcohol and other substances to self-medicate or cope with the unbearable situation (19).

The third risk factor taken into account in the FAR SEAS project is the use of other psychoactive substances, i.e., tobacco and illicit drugs (20). The prevalence of current tobacco smokers among Polish women of age 15 + is 17%, e-cigarettes smokers is 4% (21), lifetime prevalence of any drug among women from 18 to 64 years of age is 10% (the most popular illegal substances is marijuana and hashish, i.e., cannabis products –8% of surveyed women) (22). About 40% of women who drank alcohol during pregnancy also used at least one other psychoactive substance–most commonly tobacco and marijuana (23). Polydrug use during pregnancy may increase negative pregnancy outcomes and health problems for the child (24–27).

## 2. Materials and methods

The pilot methodology was based on Participatory Action Research (PAR) (28–31)—in order to build an understanding of the complexities of FASD prevention at the local and regional level, and to facilitate and evaluate community-based activities (empowering and activating local stakeholders, recruiting service providers, communication strategy, etc.). This approach facilitates capacity building in a community to promote health and solve problems; and acknowledges the fact that local knowledge is essential to achieve an accurate understanding of local problems, and to design the most adequate measures.

**FIGURE 1:**
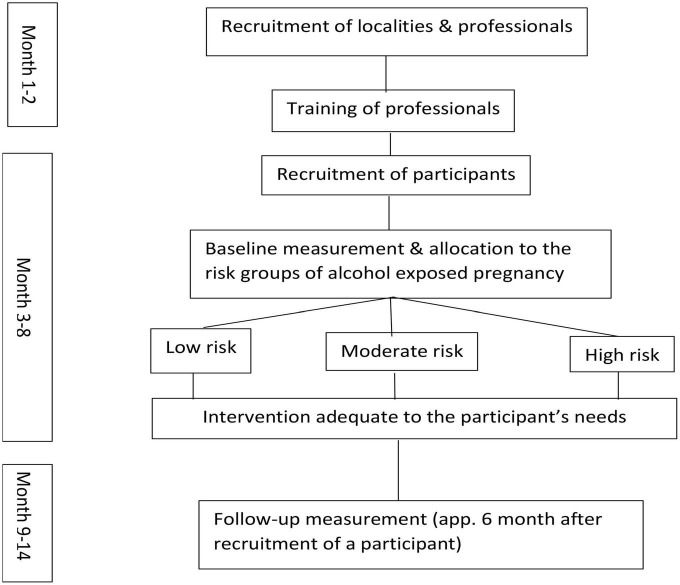
The flow chart of the pilot project.

The general overview of the pilot project activities is presented on the flow chart (Figure 1) and described in details below. The local community multi-professional teams were recruited and trained in Spring 2021. Recruitment of the participating women took place between June and December 2021 and the follow up data collection from January to July 2022.

The study was conducted in accordance with the Declaration of Helsinki, and approved by the Ethics Committee of State Agency for Prevention of Alcohol Related Problems (Poland). Informed consents were obtained from all subjects involved in the study (from local professionals and participants).

### 2.1. Target population

Females from 15 to 49 years of age were eligible to participate, whether pregnant or not pregnant, but without recognized infertility. Several entry points were planned to reach different groups of participants:

• Primary Health Care Units (PHCU)—for women of childbearing age (including pregnant women) coming for a routine visit.
• Gynecological centers–for women visiting gynecologists/obstetricians for checkups or at the beginning of pregnancy.
• Public mental health centers—for women with mental health disorders.
• Addiction treatment centers—for women with alcohol or drug use problems.
• Social Service Centers—for women with alcohol problems and/or psychosocial risk factors, as well as women whose children are at risk of being taken into foster care because of their mothers’ alcohol problems.
• Special centers for youth with behavioral disorders—for girls and young women (aged 15 +) who are at risk of unplanned teenage pregnancy.
• NGOs/Abstinence organizations—for women who either have/had alcohol problems themselves, or who live in families with alcohol-related problems.

In line with the PAR approach (28–31), a non-probabilistic sampling method was applied participants. The local staff (service providers) guided by the FAR SEAS training, were encouraged to invite all women in childbearing age (15–49 years old) they were in touch with via any of the entry points mentioned above. The invitation to the project included providing information about the project (orally and by written form) and obtaining informed consent to participate.

In practice, the local specialists had a freedom in selecting the project participants. Qualitative data collected *post hoc* indicated that they adopted different strategies–some limited recruitment to their clients, and others–actively sought participants, also using their non-professional contacts in family and among friends. This flexible procedure was chosen as the most realistic and feasible approach, taking into account variability of local contexts, recruiting professionals, and entry points in the pilot project, as well as the difficulties in accessing the study target population.

### 2.2. Professionals

The intention of FAR SEAS was to create five independent multi-professional teams to work in the five communities within the region. As each local context is slightly different, with established leaders occupying a variety of roles, the first and vital step in creating the new local interdisciplinary teams was to invite representatives of local authorities to cooperate with the project, in order to identify key local institutions, and specialists who could form a team. After getting the service managers and planners on board, representatives from Primary Health Care (PHC), gynecology/obstetric centers, social services, mental health, addiction centers and other professions were invited to join each of the local teams. Their tasks included:

• Recruitment of participants;
• Collecting baseline data and assigning women to the appropriate intervention group (low, moderate, or high risk of alcohol exposed pregnancy);
• Providing services adequate to the allocation and/or referring participants to another specialist within the local team;
• Follow-up assessment of participants (approximately 6 months after recruitment).

Remuneration was offered to the providers for each activity within the project.

### 2.3. Training

All professionals were invited to participate in the online training aimed at building capacity to work within the local interdisciplinary team in the FAR SEAS pilot. The topics of the 7-h training course included: Risks and consequences of the consumption of alcohol and other drugs during pregnancy; skills and tools to address and prevent alcohol-related harm in pregnant and child-bearing age women; common understanding of the work to be done, data collection coordination and good cooperation and referral pathways within the local team. Volunteering staff members had the opportunity to attend additional training (11 h) on motivational interviewing (MI) and the CHOICES program (31–33) aimed at preparing individuals to work with participants with moderate or high risk of alcohol exposed pregnancy.

### 2.4. Measures

After giving informed consent to participate in the project, participants were invited to have a structured computer assisted personal interview (CAPI) to determine their current pregnancy status (yes/no/trying to get pregnant), socio-demographic characteristics (age, education, work, family, and housing situation) and risk factors for alcohol use during pregnancy. The interview lasted approximately 20–30 min and no compensation was offered for baseline and follow up assessments, nor for the participation in interventions.

For women who were not pregnant, these risk factors included: (1) Risky alcohol consumption—measured with the AUDIT-*C*-test (34) and cut-off point 4. Where the score was 4+, the entire AUDIT test (35) was applied; and (2) Contraception use in the past 3 months—measured with two questions: (1) “Are you sexually active?” and if yes, and (2) “What contraceptive method do you normally use?” with a range of options [None, Condoms, Birth Control Pills, Vaginal Ring (NuvaRing), Contraceptive patch, Emergency Contraception (e.g., morning-after pill), Contraceptive progestin injection (medroxyprogesterone acetate/e.g., Depo-Provera Shot), IUD (intrauterine devices/coil), Birth control implant (e.g., Implanon, Nexplanon), Other]. The answers were dichotomized into “no” and “yes” use of contraception and sexually non-active participants were excluded from analysis.

Women who were pregnant at the time of recruitment, were asked the AUDIT-C questions first, in reference to the last 3 month before getting pregnant; and, then, the same questions but on the period during pregnancy. Score of 4+ before pregnancy was interpreted as a risk factor for alcohol use at least in the first trimester, and any alcohol use during pregnancy was an indicator of risk.

All participants were asked about current use of (a) cigarettes and (b) other drugs (psychoactive substances, sedatives or sleeping pills). Women who were not pregnant could choose one of four answers (Never used; I used to use it, but now I don’t use it; I use occasionally; I use regularly). Pregnant women had two more options: I used to smoke, but now I do not smoke because of the pregnancy; I have reduced smoking since being pregnant.

Psychosocial risk factors included depressive symptoms measured with the PHQ-9 (36–38). Moderate or higher severity of symptoms (score 10 +) was interpreted as the risk factor. Screening for domestic violence was based on the questionnaire “Assessment of the family situation in terms of violence” (39), and a positive answer to any of the 9 questions asking about different forms of physical, psychological or economical violence (e.g., “Has your partner or someone close to you ever behaved this way toward you?”: “pushed, tugged, pulled hair”; “humiliated/criticized”), was taken as an indicator of risk.

The use of services was measured with one question: “In the last 3 months, have you had any advice/consultation with a GP/nurse, gynecologist/midwife, Social worker, Addiction therapist, Psychologist/psychiatrist?” For each specialist the respondent indicated never, 1–2 times or 3 times or more.

The same questions were asked in the follow-up session approximately 6 months after recruitment. Alcohol, contraception, and service-use were the key outcome measures, and psychosocial risk factors (cigarette and drug use, depressiveness and domestic violence)—secondary outcomes.

### 2.5. Allocation to the risk group and interventions

Based on the screening results, participants were allocated to a low-, moderate- or high-risk group for risk of having a baby with FASD (Table 1). To do this, the local staff member(s) were instructed to follow the criteria:

**TABLE 1:** Risk groups allocation criteria.

Level of risk	Not pregnant	Pregnant
Low	AUDIT C < 4 and no PSR^1^	No drinking^2^ and no PSR
Moderate	AUDIT C ≥ 4 and no PSR or AUDIT C < 4 and PSR positive	Drinking and no PSR or No drinking and PSR positive
High	AUDIT C ≥ 4 and PSR positive or AUD (AUDIT ≥ 7)	Drinking

^1^PSR = Psychosocial risks.

^2^No drinking = A woman is an abstinent or drank moderately before pregnancy and stopped drinking as soon as she planned pregnancy or learned about pregnancy.

1. Low risk means: (a) if a participant is pregnant—abstinent since before pregnancy OR before pregnancy used to drink moderately (AUDIT-C score <4) and stopped drinking as soon as she learned about pregnancy; and (b) if not pregnant—no hazardous drinking and use of contraceptive measures.
2. Moderate risk means: (a) if a participant is pregnant—risky alcohol consumption (AUDIT-C score 4 +) in the past (before pregnancy) AND/OR presence of significant psychosocial risk factors (depressiveness and/or violence, and/or economic challenges, and/or use of drugs); and (b) if not pregnant—risky alcohol consumption AND/OR psychosocial risk factors including no use of contraceptive measures.
3. High risk means: (a) if a participant is pregnant—current alcohol use; and (b) if not pregnant—alcohol use disorders.

Despite the outcome according to these guiding criteria, the final decision about allocation and the intervention offered to the individual client, was always made by the local team member(s) based on their professional experience and any additional information they might have (e.g., from the contacts with a client prior to the FAR SEAS project).

The local teams were encouraged to follow up the screening by offering the intervention adequate to a client’s needs (1):

• To participants at low risk of alcohol exposed pregnancy—brief feedback to support their attitude and underline the importance of alcohol abstinence during pregnancy.
• To those in the moderate-risk group—activities providing the opportunity for safe discussion about reproductive health, contraception, pregnancy, alcohol use and related issues in a form of brief intervention (40, 41) and/or 1 to 4 individual motivational sessions (according to the client needs) aimed at changing at least one of the risky behaviors: alcohol use and/or sex without contraception [based on: Project CHOICES (31–33)].
• To those in the high-risk group–who need special support to deal and cope with their individual risk factors that make them especially vulnerable for giving birth to a child with FASD—supportive, specialized services, i.e., individual motivational sessions and/or referral to specialists in addiction therapy, psychotherapy, medicine etc., according to individual needs.

Typically, the interventions were provided in the same setting in which a participant was recruited, except for visits in a participant’s home. No compensations on retention in the project was offered to participants.

The intervention period started immediately after the recruitment of a participant. Usually, the first step, was to provide feedback on the screening results and, in the case of women from the low risk group—it was often the sole form of intervention. If more intensive measures were needed, the intervention period could be extended to a maximum of 6 months, although it usually lasted about 2–3 months, depending on the type of services and the availability of the participant. For example, some participants did not immediately decide to participate in sessions based on the CHOICES project, or returned after a longer absence for a referral to a specialist.

**FIGURE 2:**
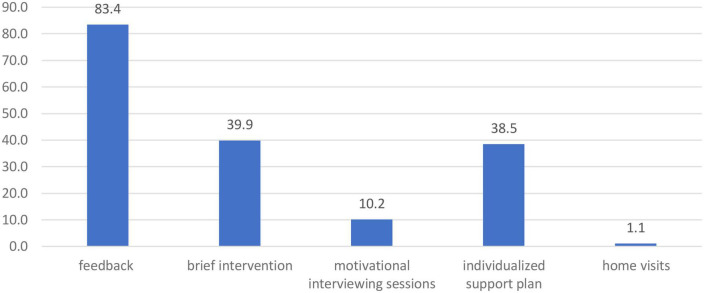
The percentage of participants (*n* = 441) who received a given form of intervention within the project.

In general, interventions that the local specialists proposed to the participants covered the full spectrum of possibilities provided for in the project (Figure 2). For women from the moderate risk group, the next step (after feedback) was a brief intervention (in most cases—covering two meetings 1 or 2 weeks apart) or motivational interview sessions aimed at reducing risky drinking and/or encouraging use of contraception. In average the number of motivational sessions based on the CHOICES approach for one participant was 1.9 but, as the final decision about the extent of the support needed by a particular participant was made individually by a local specialist, in two cases of women at high-risk of PAE, support was extended to 5 and 6, compared to 4 sessions initially planned.

Individualized support plans were developed for *n* = 170 women including, individual motivational sessions and referrals to specialists (psychologist, gynecologist, midwife, addiction therapist, and others). The individualized offer for 5 participants also included home visits.

### 2.6. Data analysis

Analyses were performed using IBM SPSS Statistics 28.0. In case of the continuous variable—participants’ age, the *F*-test was adopted to make cross-group comparisons. All other data were presented as frequencies (Freq) for categorical variables, and the chi-squared test was adopted for comparisons between groups. The evaluation of improvements in desired outcomes was based on the non-parametric sign test, testing differences between pairs of observations before and after intervention. The sizes of groups with positive values at baseline and follow-up were compared in the entire sample, and in the sub-groups differentiated by the level of risk of having a baby with FASD. In all tests, a *p*-value of <0.05 was considered statistically significant. To illustrate and facilitate interpretation of the statistically significant results, the coefficients of variation between measures were calculated according to the formula:


Freq(followup)-Freq(baseline)]/Freq(baseline)*100.

## 3. Results

### 3.1. Characteristics of professionals

Initially, 30 specialists from 5 different sites–two towns (Płock, and Radom) and 3 districts of Warsaw City–agreed to participate in the project. Teams from two of the Warsaw districts withdrew from the project just before or immediately after the initial training. Therefore, an additional team (5 professionals: 2 social workers, 1 psychologist, 1 pedagogue, 1 addiction therapist) from another town–Pruszków–was recruited. In the end, 31 professionals participated in the initial training and 25 of these undertook project activities. Most of them were social workers and/or family assistants–providing practical support to the family in consultation with the social worker (42) (4 in Plock, 3 in Radom, 2 in Ursus). All local teams included at least one psychologist (2 in Radom) and one addiction therapist. The Płock team included also the pedagogue and the member of the abstainers’ association. No one medical doctor was involved in any of the teams but in Radom one midwife and in Płock one nurse/midwife was engaged.

### 3.2. Characteristics of participants

The final sample consisted of *N* = 441 women recruited into the project by local staff. The majority of participants were recruited via social services (77.3%), followed by addiction therapy (5.7%) and health care facilities (2.5%). All other participants (14.5%) were recruited via personal contacts of local staff members (all profiles) or NGOs. Among the recruited participants, 9.5% (*N* = 42) were pregnant at the time of recruitment.

The majority of participants were recruited in Płock (61.7%), followed by Radom (34%). After screening, 69.6% of the total sample were allocated to the low-risk group (*N* = 307); 22.67% to the moderate-risk group (*N* = 100); and to the high-risk group 7% (*N* = 31) (Table 2).

**TABLE 2:** Recruitment of participants and their allocation to alcohol exposed pregnancy risk group by the entry site location.

	The risk of alcohol exposed pregnancy (*n*)				All (*n*)	Pregnant women (*n*)
**Location**	**Low**	**Moderate**	**High**	**Not assessed**		
Warsaw-Ursus	1	1	0	2	4	1
Płock	196	69	7	0	272	23
Radom	98	28	23	1	150	10
Pruszków	12	2	1	0	15	8
All	307	100	31	3	441	42
%	69.61%	22.68%	7.03%	0.06%	100%	9.52%

The mean age of participating women was 33 years (SD = 8.46), ranging from 16 to 49 years (2 participants were underage—16 and 17 years old). Regarding other socio-demographic characteristics: almost half of the sample were married or in a constant relationship (49%); 75% had secondary or higher education; and over 60% were employed. Significant differences in terms of socio-demographic features between risk groups have been noted (Table 3): in mean age (the low-risk group being significantly older); occupational status (employment rate decreased with group risk); marital and living status (constant relationships and living with a husband/partner and a child/children were most prevalent in low-risk group).

**TABLE 3:** Baseline characteristics of participants by their allocation to alcohol exposed pregnancy risk group.

	Low risk (*n* = 307)	Moderate risk (*n* = 100)	High risk (*n* = 31)	*p*
Mean age (range)	34.10 (16–49)	31.57 (18–49)	30.87 (17–43)	0.008^1^
**Education**				
Primary	11.1%	13.6%	29.0%	0.118^2^
Vocational	9.5%	13.6%	9.7%	
Secondary	37.0%	41.7%	35.5%	
Tertiary	41.3%	30.1%	25.8%	
**Occupational status**				
Employed	66.2%	51.5%	38.7%	<0.001
Student	16.8%	25.0%	11.8%	0.369
Unemployed	41.6%	33.3%	58.8%	
Health problems	9.9%	10.4%	17.6%	
Child care	31.7%	31.3%	11.8%	
**Marital status**				
Married/constant relationship	56.7%	36.9%	22.6%	0.002
Single	28.9%	46.6%	51.6%	
Divorced/separation	12.5%	14.6%	22.6%	
Widow	1.3%	1.9%	3.2%	
**Living with …**				
Alone	4.9%	1.9%	9.7%	<0.001
Partner/husband and children	43.6%	27.2%	22.6%	
Partner/husband	19.7%	21.4%	9.7%	
Other relatives	12.1%	28.2%	35.5%	
A child/children	15.7%	19.4%	12.9%	
Other	3.9%	1.9%	9.7%	
**Psychosocial risk factors**				
Domestic violence	5.4%	12.6%	38.7%	<0.001
Depressive symptoms	1.7%	13.5%	39.3%	<0.001
Current cigarette use	26.1%	65.3%	86.2%	<0.001
Current drug use	1.6%	7.8%	19.4%	<0.001
**Not pregnant:**				
No contraception use (not pregnant)	42.9%	54.3%	60.0%	0.067
Risky alcohol use (not pregnant)	1.1%	20.4%	63.0%	<0.001
**Pregnant when recruited**	*N* = 29	*N* = 9	*N* = 4	
Risky alcohol use before pregnancy	*N* = 0	*N* = 1	*N* = 4	
Any alcohol use during pregnancy	*N* = 1	*N* = 2	*N* = 1	

^1^*F*-test.

^2^Here and in the following columns chi square test was used.

As expected, given the allocation process, the prevalence of psychosocial factors increasing the risk of alcohol use during pregnancy (being a victim of domestic violence, elevated risk of depression, psychoactive substance use, and risky drinking when not pregnant) were the lowest in the low-risk group and the highest in the high-risk group. The only risk factor for which the statistical test of inter-group differences did not reach the significance level was the use of contraceptive measures. Across all groups, nearly half of the participants (46.9%) did not use contraception.

### 3.3. Follow-up results

At follow up, data were collected from 411 participants (93.2% of the sample).

Among the 42 women who were pregnant at the time of the recruitment, *n* = 5 reported risky alcohol consumption before pregnancy and *n* = 3 reported any alcohol use during pregnancy (Table 3). None of the *n* = 7 women who were still pregnant at the time of the follow-up measurement drank alcoholic beverages. These differences were not tested statistically.

In the entire sample of not pregnant women, self-reported risky alcohol consumption dropped at follow-up after 6 months indicating the coefficient of variation = −81.25, and contraception use increased by 15 percentage points (Table 4).

**TABLE 4:** Changes in the risk factors of FASD in a child between the baseline and the follow-up, assessed with the sign test (entire sample).

		*N*	*p*	Coefficient of variance
Risky alcohol use	Positive change^1^	53	<0.001	−81.25
	Negative change	1		
	No change	314		
Contraception use	Negative change	12	<0.001	14.59
	Positive change^2^	39		
	No change	295		
Current cigarette use	Positive change^3^	35	<0.001	−18.99
	Negative change	5		
	No change	369		
Current drug use	Positive change^4^	14	0.004	−63.16
	Negative change	2		
	No change	397		
Domestic violence	Positive change^5^	25	<0.001	−100
	Negative change	0		
	No change	376		
Depressive symptoms	Positive change^6^	80	0.012	−53.57
	Negative change	18		
	No change	306		
Consultation with a GP/nurse	Negative change	76	0.750	
	Positive change^7^	81		
	No change	246		
Consultation with a gynecologist	Negative change	57	<0.001	39.19
	Positive change^7^	105		
	No change	242		
Consultation with a social worker	Negative change	33	0.903	
	Positive change^7^	35		
	No change	328		
Consultation with an addiction therapist	Negative change	9	0.607	
	Positive change^7^	6		
	No change	383		
Consultation with a psychologist/psychiatrist	Negative change	25	1.000	
	Positive change^7^	24		
	No change	346		

^1^Follow up risky alcohol use <Baseline risky alcohol use.

^2^Follow up contraception use >Baseline contraception use.

^3^Follow up cigarette use <Baseline cigarette use.

^4^Follow up drug use <Baseline drug use.

^5^Follow up domestic violence <Baseline domestic violence.

^6^Follow up depressive symptoms <Baseline depressive symptoms.

^7^Follow up consultations with a specialist >Baseline consultations with a specialist.

Changes in all other risk factors were assessed in the entire sample (regardless of the pregnancy status at the time of recruitment). Positive outcomes (decreases) were observed for all psychosocial risk factors: current cigarette and drug use, domestic violence, and depressive symptoms. No changes occurred in the frequency of visits to specialists, except for visiting a gynecologist, which increased by 39.19 percentage points (Table 4).

The analysis of changes in FASD risk factors in the low-risk group of participants (Table 5) indicated a significant increase in contraception use and visits to a gynecologist (by 9.49 and 32.14 percentage point, respectively). At the same time, the number of current cigarette smokers decreased in this group by 22.22 percentage points.

**TABLE 5:** Changes in the risk factors of FASD in a child between the baseline and the follow-up in the low-risk group, assessed with the sign test.

		*N*	*p*	Coefficient of variance
Risky alcohol use	Positive change^1^	7	0.070	
	Negative change	1		
	No change	247		
Contraception use	Negative change	10	0.037	9.49
	Positive change^2^	23		
	No change	204		
Current cigarette use	Positive change^3^	19	<0.001	−22.22
	Negative change	3		
	No change	264		
Current drug use	Positive change^4^	3	0.625	
	Negative change	1		
	No change	285		
Domestic violence	Positive change^5^	5	0.063	
	Negative change	0		
	No change	272		
Depressive symptoms	Positive change^6^	5	0.774	
	Negative change	7		
	No change	268		
Consultation with a gynecologist	Negative change	44	0.015	32.14
	Positive change^7^	71		
	No change	170		

^1^Follow up risky alcohol use <Baseline risky alcohol use.

^2^Follow up contraception use >Baseline contraception use.

^3^Follow up cigarette use <Baseline cigarette use.

^4^Follow up drug use <Baseline drug use.

^5^Follow up domestic violence <Baseline domestic violence.

^6^Follow up depressive symptoms <Baseline depressive symptoms.

^7^Follow up consultations with a specialist >Baseline consultations with a specialist.

In the moderate-risk group, significant changes occurred in all outcome variables in the desired direction (Table 6).

**TABLE 6:** Changes in the risk factors of FASD in a child between the baseline and the follow-up in the moderate-risk group, assessed with the sign test.

		*N*	*p*	Coefficient of variance
Risky alcohol use	Positive change^1^	35	<0.001	−94.59
	Negative change	0		
	No change	55		
Contraception use	Negative change	1	0.006	25.00
	Positive change^2^	11		
	No change	76		
Current cigarette use	Positive change^3^	13	0.007	−17.46
	Negative change	2		
	No change	82		
Current drug use	Positive change^4^	6	0.031	−75.00
	Negative change	0		
	No change	92		
Domestic violence	Positive change^5^	10	0.002	−100.00
	Negative change	0		
	No change	88		
Depressive symptoms	Positive change^6^	11	0.006	−83.33
	Negative change	1		
	No change	80		
Consultation with a gynecologist	Negative change	9	0.007	64.29
	Positive change^7^	26		
	No change	58		

^1^Follow up risky alcohol use <Baseline risky alcohol use.

^2^Follow up contraception use >Baseline contraception use.

^3^Follow up cigarette use <Baseline cigarette use.

^4^Follow up drug use <Baseline drug use.

^5^Follow up domestic violence <Baseline domestic violence.

^6^Follow up depressive symptoms <Baseline depressive symptoms.

^7^Follow up consultations with a specialist >Baseline consultations with a specialist.

In the high-risk group, risky alcohol consumption dropped by 61.11 percentage points, depressive symptoms by 63.64, and domestic violence by 100 (Table 7).

**TABLE 7:** Changes in the risk factors of FASD in a child between the baseline and the follow-up in the high-risk group, assessed with the sign test.

		*N*	*p*	Coefficient of variance
Risky alcohol use	Positive change^1^	11	<0.001	−61.11
	Negative change	0		
	No change	12		
Contraception use	Negative change	1	0.219	
	Positive change^2^	5		
	No change	15		
Current cigarette use	Positive change^3^	3	0.250	
	Negative change	0		
	No change	23		
Current drug use	Positive change^4^	5	0.219	
	Negative change	1		
	No change	20		
Domestic violence	Positive change^5^	10	0.002	−100.00
	Negative change	0		
	No change	16		
Depressive symptoms	Positive change^6^	7	0.016	−63.64
	Negative change	0		
	No change	19		
Consultation with a gynecologist	Negative change	4	0.388	
	Positive change^7^	8		
	No change	14		

^1^Follow up risky alcohol use <Baseline risky alcohol use.

^2^Follow up contraception use >Baseline contraception use.

^3^Follow up cigarette use <Baseline cigarette use.

^4^Follow up drug use <Baseline drug use.

^5^Follow up domestic violence <Baseline domestic violence.

^6^Follow up depressive symptoms <Baseline depressive symptoms.

^7^Follow up consultations with a specialist >Baseline consultations with a specialist.

No negative effects of the interventions were noted in any of the sub-groups, nor in the sample as a whole.

## 4. Discussion

The evaluation showed positive results of the interventions conducted in terms of the change in the key targeted behavior, i.e., a reduction in the percentage of women who drink alcohol in a risky manner. Significant decrease in psychosocial risk factors (current cigarette and drug use, depressiveness, and reporting domestic violence) were also observed. Positive changes were also noted, but on a smaller scale, in terms of the increasing percentage of women using contraception and visiting specialists in gynecology.

These positive results were obtained in spite of the COVID-19 pandemic restrictions introduced in Poland in 2020 and unprecedented conditions of health work at that time. Due to the COVID-19 pandemic, health professionals in particular were overstretched and limited their work with patients to telephone counseling. It is likely that this was one of the main reasons for the low levels of medical professionals participating. But, as indicated the unofficial recruitment talks, there were also other barriers, such as, e.g., those reported in the Danish study (43): poor confidence in navigating between health and social care systems, fear of breaking the professional-patient alliance when touching the alcohol consumption issues in antenatal care or reporting to the social services.

An absence or scarcity of medical professionals in the local teams created additional challenges for the other professionals active in the project. However, the positive outcomes of evaluation indicate that the implementation of FASD prevention initiatives by professionals in the social care system, among others, is feasible and can be effective. This conclusion from our study seems particularly important and interesting due to the limited number of publications on the activities of social workers in the area of preventing alcohol consumption during pregnancy. Studies on the effectiveness of brief interventions or counseling are usually conducted in healthcare settings (44, 45). The community approach is applied only to work in the case management paradigm, with women proven to be at high risk for drinking during pregnancy (46, 47). Social workers’ role is discussed, either as an element of much broader multi sectoral system approach (48, 49) or as recipients of professional trainings (50, 51).

Although our project is a pilot–focused on feasibility, process, and reaching different groups, rather than focusing purely on the effectiveness of a single prescriptive approach (52, 53), it provides the impetus for further exploration of the outcomes of FASD preventive interventions by social workers. In particular, it is notable that the professionals in our pilot project had considerable freedom of action, as indicated by fact that they made decisions on the scope of intervention for individual participants on their own or after consultations with other members of the local team. These decisions concerned, for example, the number of individual motivational sessions to conduct with each woman. The American experience of the CHOICES program shows that, depending on the individual recipient, working with 1, 2, or 4 sessions may be effective (31–33). Because our specialists worked with very different women (pregnant and not pregnant; occasional, risky or problem drinkers; with or without other psychosocial problems), they independently decided how many sessions to conduct in a given case. This highly individualized, tailored approach was probably one of the biggest strengths of the FAR SEAS project pilot, in line with the recommendations from the systematic review of FASD prevention programs (54). It could be concluded that the FAR SEAS pilot study tested the scale-up of a new flexible intervention approach, addressed at a broad target group of women, but tailored to the different women’s characteristics and situations.

The specific profiles of the intervention teams, relying largely on social workers, also determined the profile of participants reached, among which there were few pregnant women. On the other hand, the rate of people at higher risk of drinking alcohol during pregnancy in our sample (30% classified to moderate and high risk groups) was higher than in the general population [based on the data indicating that 22% of women of child bearing age in Poland have had at least one Risky Single Occasion Drinking—RSOD episode in the past 12 months (55)].

The small number of pregnant participants in the study does not allow conclusions to be drawn on the effectiveness of the FAR SEAS approach during pregnancy specifically (even in a situation where we did not record any cases of alcohol consumption among participants who were pregnant at the time of last study visit). However, our findings do suggest positive influences of the interventions on the participants taken as a whole, especially those who drink alcohol at risky level and/or present other psycho-social risk factors of drinking alcohol during pregnancy, and therefore at higher risk of giving birth to a child with FASD (i.e., our moderate-risk group).

Given the importance of being able to target the intervention to the group most in need of support, i.e., women most at high risk of alcohol exposed pregnancy, the result indicating significant reductions in risky alcohol consumption among the high-risk subgroup is very promising. It is also important that the interventions offered to this group within the FAR SEAS project had positive effects on their domestic situation (reduced violence) and mood (depressive symptoms). However, more intensive or different interventions, or the engagement of other specialists (probably, linking to health professionals) are needed to improve their reproductive health (i.e., contraception use, visiting a gynecologist), and to reduce other psychoactive substance use in pregnancy.

The improvement of some health behaviors (use of contraceptive measures, visits to a gynecologist, reduced cigarette smoking) in the low-risk group, suggests a need for basic health education for the general Polish population, especially among women in childbearing age.

The applicability of the study results to different populations and contexts may be limited by the uneven spread of participants across the different geographical and urban sites. An unstable social and medical situation at the time of the study made the recruitment of local stakeholders and individual specialists very difficult. Out of the six local teams that signed up for the project, only 4 teams were active, including 2 which were active only at a minimal level. In two other locations of the project (Płock and Radom), interdisciplinary teams of specialists (social workers, psychologists, addiction therapists, midwifes) were established to implement all the planned activities. Both highly active teams were led by people evidently well predisposed to this role: engaged, well organized, oriented to problem-solving and communicative (56, 57). In Radom the leader from social service center was appointed by the local authorities, while in Płock—one of social workers volunteered to this role. These teams managed to recruit and attend over 400 participants, the vast majority (93%) of whom also took part in the final measurement, which is a strength of the current data set. It is possible that a lack of good coordination and leadership was one of the reasons for the limited uptake and success of the pilot in the two other teams.

One other limitation of the study, which is encountered in many alcohol prevention interventions, is the reliance on self-reported data for alcohol use, which may result in a social desirability bias (which we can expect to be especially present among pregnant women in the follow-up sessions). In addition, the freedom of local staff in selecting the project participants, although reflecting natural context of their work, may be considered a limitation of the study. It is possible that, consciously or subconsciously, they introduced a bias by inviting women they feel they can more easily work with, who may not be those most in need of the intervention. This indicates the need of more rigorous training and enforcement of research experiment standards in future studies.

## 5. Conclusion

The positive results of the pilot project indicate the feasibility and validity of implementing multi-center regional FASD prevention through inter-disciplinary teams at the community level. The most prominent changes in the prevalence of risky alcohol consumption were observed in the moderate risk group indicating potentially high returns on investment of addressing FASD prevention and interventions to this group; which should not be overlooked, despite the priority of focusing on women at high risk of alcohol exposed pregnancies. The results suggest an opportunity to build on the receptivity of women in the moderate risk group to prevention and advice, and that delivering timely interventions may prevent women from developing more risky behaviors leading to alcohol exposed pregnancies. Dissemination of FASD prevention at the local level requires the involvement of local authorities and directors of key institutions and coordinated work in a multidisciplinary team, which is one of the key facilitators of good implementation and sustainability of FAS/FASD prevention.

The main learning points and findings from the FAR SEAS pilot study have been fed forward into the FAR SEAS Guidance, which comprises 22 evidence-based recommendations, validated through international expert consensus, aimed at reducing alcohol consumption in women of childbearing age, particularly in pregnant women.

## Data availability statement

The raw data supporting the conclusions of this article will be made available by the authors, without undue reservation.

## Ethics statement

The study involving humans was approved by the Ethics Committee of State Agency for Prevention of Alcohol Related Problems (Poland). The study was conducted in accordance with the local legislation and institutional requirements. Written informed consent for participation in this study was provided by the participants’ legal guardians/next of kin.

## Author contributions

KO-K, LS-G, FB, CG, and ES contributed to conception and design of the study. KO-K and MZ-S organized the database. KO-K, SG, and MZ-S performed the statistical analysis. KO-K, FB, LS-G, and CB wrote the first draft of the manuscript and sections of the manuscript. FB, SM, KO-K, and LS-G supervised the project implementation. FB and SM administered the project. FB, SM, and KO-K acquired funding for the project. All authors contributed to manuscript revision, read, and approved the submitted version.
